# A Rare Case of Endometriosis of the Small Bowel

**DOI:** 10.1155/2021/6676855

**Published:** 2021-05-08

**Authors:** Moisés R. Zepeda, Su K. Win

**Affiliations:** ^1^Department of Pathology, Alhambra Hospital Medical Center, 100 S. Raymond Avenue Alhambra, CA 91801, USA; ^2^Department of Pathology, Louisiana State University Health Sciences Shreveport, 1501 Kings Highway Shreveport, LA 71103, USA

## Abstract

Endometriosis of the small bowel is a rare clinical event. The clinical condition presents with vague abdominal symptoms and is usually not diagnosed acutely, unless clinicians have a high index of suspicion. Most patients are diagnosed after multiple clinical encounters. We present a case of endometriosis causing small bowel obstruction diagnosed postsurgically.

## 1. Introduction

Endometriosis is defined as the presence of normal endometrial tissue located outside of the endometrial cavity and myometrium [1, 2]. It is not an uncommon condition, as it affects about 6-10% of reproductive age women. The common sites of endometriosis, in descending order, include the ovaries, uterine ligaments, rectovaginal septum, cul-de-sac, pelvic peritoneum, bowel, and appendix [1, 2]. According to Wolthuis et al., intestinal endometriosis accounts for 3-37% of intestinal cases, and the small bowel is involved in only about 10% of all intestinal endometriosis cases [3]. In turn, small bowel obstruction due to endometriosis occurs in less than 7% of the cases. And less than 1% of these cases need surgical resection. Saleem et al. stated that different studies have reported the incidence of endometriosis in the appendix to be from 0.2% to 1.3% [4]. These statistics confirm that cases of endometriosis causing small bowel obstruction, with concomitant appendiceal endometriosis, are an extremely rare clinical event. We therefore present a case report of small bowel obstruction secondary to endometriosis. It is important to note that most cases are diagnosed postsurgically. Therefore, since our case was diagnosed postsurgically only after histopathological examination of small bowel and appendiceal tissues, our description and presentation will delineate the histopathological findings.

## 2. Case Presentation

**Figure 1:**
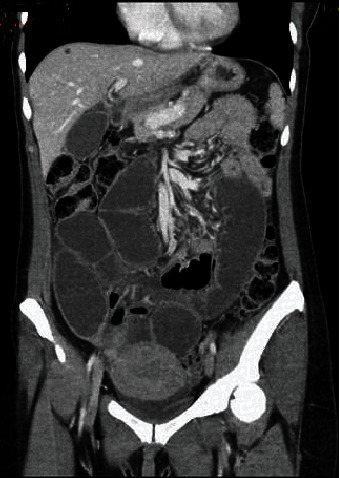
Abdominal computed tomographic scan showing dilated small bowel loops.

**Figure 2:**
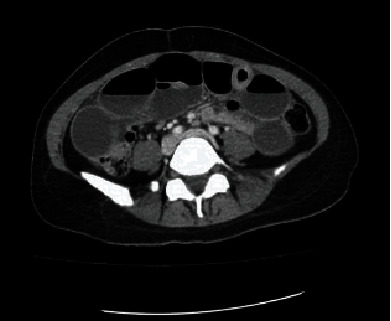
Abdominal computed tomographic scan showing dilated small bowel loops with air fluid levels.

A 41-year-old female presented to the emergency department (ED) with a chief complaint of abdominal pain. The patient reported having chronic abdominal pain for more than two months with worsening pain in her right lower abdomen. On physical examination, pertinent laboratory studies, and radiological studies, a clinical diagnosis of small bowel obstruction was determined by the ED physician. The salient radiological studies, abdominal X-ray and Computed Tomography (CT) scan of her abdomen and pelvis, showed short segments of small bowel wall thickening and dilated loops of bowel, which were consistent with a clinical diagnosis of small bowel obstruction (Figures 1 and 2). A surgical consultation was ordered, and the patient underwent an exploratory laparotomy. The attending surgeon confirmed the radiological diagnosis of a small bowel obstruction and a further finding of suspected acute appendicitis. A large portion of distal ileum was resected along with the appendix.

The patient's chart was reviewed postsurgically, and it was revealed that she first presented to the ED with left lower abdominal pain six years prior to admission. During that visit, the patient reported dysuria and menorrhagia. At that same visit, an ultrasound study revealed a small mildly complex right ovarian cyst measuring 3.6 × 2.2 centimeters. The patient was treated conservatively and discharged home. Subsequent to this first ED visit, the patient presented multiple times to the ED with similar complaints of abdominal pain and continued to be treated conservatively with pain medications.

## 3. Pathology

The specimen consisted of a 23-centimeter length of small bowel varying in external diameter from 4 to 6 centimeters. The bowel mucosa was erythematous with focal areas showing edema. The bowel wall showed multiple loops of adherent serosa and multifocal areas of thickened muscularis externa, with a maximal thickness of 2.2 centimeters. The appendix was also submitted to our laboratory as a separate specimen. The appendix was grossly unremarkable.

**Figure 3:**
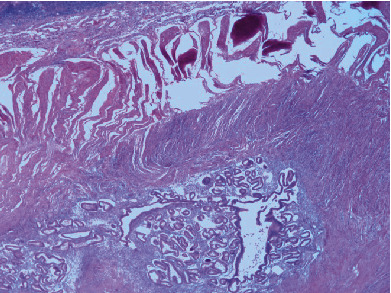
Endometriotic foci in the muscularis externa of the terminal ileum (H&E, 2x).

**Figure 4:**
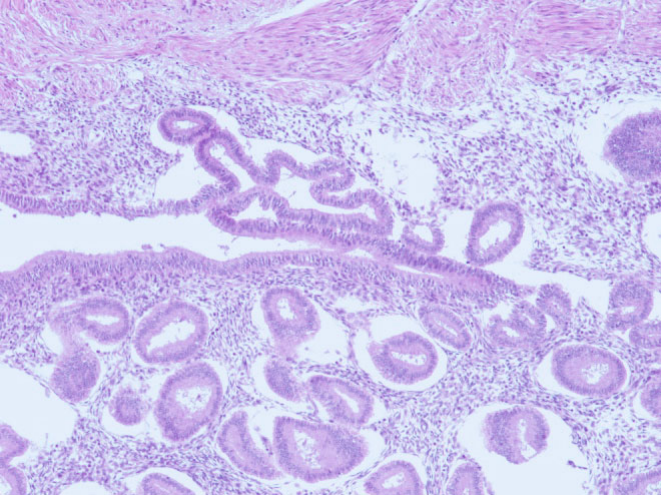
Endometriotic foci in the muscularis externa of the terminal ileum (H&E, 10x).

**Figure 5:**
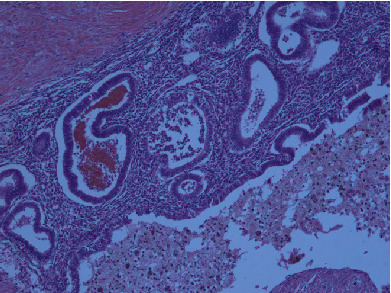
Endometrial glands with hemorrhage and stroma (H&E, 10x).

**Figure 6:**
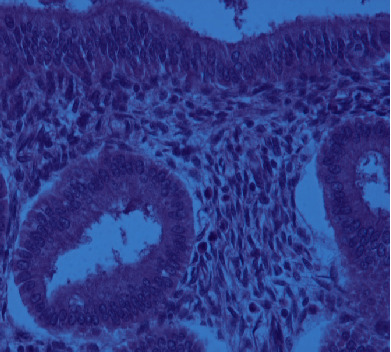
Endometrial glands and stroma (H&E, 40x).

**Figure 7:**
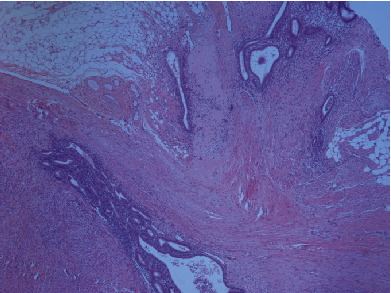
Endometrial glands and stroma within mesoappendix and wall (H&E, 4x).

Histologic Hematoxylin and Eosin (H&E) sections of the small intestine demonstrated multiple areas of endometrial tissue within the muscularis externa (Figures 3 and 4). The microscopic findings clearly showed benign endometrial glands with associated endometrial stroma, and some areas also showed active bleeding and hemosiderin-laden macrophages (Figures 5 and 6). Sections of the appendix also showed endometrial glands and stroma. The endometriotic lesions were identified within the mesoappendix and appendiceal wall (Figure 7).

## 4. Discussion

Multiple authors have proposed multiple theories on the etiology and pathogenesis of endometriosis. These theories comprise the following: (1) the regurgitation theory of retrograde menstrual blood flow; (2) the metastatic theory in which endometrial tissues spread via blood or lymphatic channels; (3) the metaplastic development theory in which coelomic epithelium transforms into endometrial tissue; and (4) the most recent stem cell theory in which bone marrow stem cells differentiate into endometrial tissue at ectopic anatomical sites [2–4]. Regardless of which theory clinicians subscribe to, patients still suffer the clinical consequences of this disease. This is mostly due to the difficulties of establishing a noninvasive clinical diagnosis. However, promising new research is shedding new light on providing novel noninvasive diagnostic laboratory methods for confirming endometriosis [5–7].

From infertility issues to unmanageable pain, endo.metriosis causes untold morbidity, including economic and social distress, for women and their families [6–9]. That is why it is paramount to have a high index of suspicion when reproductive age women present to their healthcare providers with vague signs and symptoms of abdominal pain [10, 11]. The earlier this disease is diagnosed, then the earlier women can be offered different treatment modalities. Early intervention can mitigate catastrophic clinical outcomes, such as the small bowel resection that was experienced by our patient.
